# Fine-Scale Strontium Isotope Mapping in Eastern China (Anhui Province) and Its Application in Food Traceability

**DOI:** 10.3390/foods15010033

**Published:** 2025-12-22

**Authors:** Mei Wang, Yunlu Jiang, Xiaojing Han, Aoyu Ren, Jiahui He, Minzhen Yin, Yujiao Zhao, Huasheng Peng

**Affiliations:** 1 State Key Laboratory for Quality Ensurance and Sustainable Use of Dao-di Herbs, National Resource Center for Chinese Materia Medica, China Academy of Chinese Medical Sciences, Beijing 100700, China; mw9741@126.com (M.W.); yunlu_jiang@126.com (Y.J.); jxhan511@163.com (X.H.); ay_ren@126.com (A.R.); jiahuihe0818@163.com (J.H.); minzheny@126.com (M.Y.); 2 School of Pharmacy, Anhui University of Chinese Medicine, Hefei 230012, China; zhyj2828@163.com; 3 Key Scientific Research Base of Traditional Chinese Medicine Heritage (Institute of Chinese Materia Medica, China Academy of Chinese Medical Sciences), National Cultural Heritage Administration, Beijing 100700, China

**Keywords:** Sr isotope map, ^87^Sr/^86^Sr, origin traceability, Anhui province, China

## Abstract

Origin traceability is critical for food safety, and the strontium isotope ratio (^87^Sr/^86^Sr) has been widely used in this field due to its accuracy and stability. Establishing a regional baseline map of bioavailable ^87^Sr/^86^Sr is essential for precise traceability. However, the existing large-scale bioavailable Sr isotope map of China has low spatial resolution and uses water as the main sample, making it unsuitable for plant-derived food traceability. This study focused on Anhui, a major agricultural province in China. Based on geological lithology distribution, 149 plant samples were collected across Anhui to construct a fine-scale bioavailable ^87^Sr/^86^Sr map. The map enabled traceability of Anhui’s characteristic plant-derived foods, such as Cha (*Camellia sinensis*), Mugua (*Chaenomeles speciosa*), Fengdan (*Paeonia ostii*), Jiegeng (*Platycodon grandiflorum*), and Duohua Huangjing (*Polygonatum cyrtonema*). It provides a basis for food origin traceability in Anhui and supports market supervision of China’s geographical indication (GI) products.

## 1. Introduction

Food origin traceability, as a core technical means to ensure the authenticity of regional characteristic agricultural products, holds considerable importance for conserving these products [1]. Specifically, accurate traceability can prevent counterfeiting of geographical indication products and protect the reputation of authentic regional specialties, thereby encouraging local farmers to maintain traditional planting practices and ecological protection measures, which are key prerequisites for the long-term conservation of regional characteristic agricultural resources. With the increasing complexity of global food supply chains, the authenticity and geographical origin of food have become key concerns among various stakeholders. Regions such as the European Union have established systems such as the “Protected Designation of Origin” to provide legal protection for foods produced within specific geographical areas, thereby preserving their unique quality and reputation [2]. However, the frequent occurrence of food adulteration and mislabeling has made the development of accurate and efficient technologies for food origin identification a hotspot in food safety.

Currently, analytical methods used for food geographical origin traceability primarily include spectroscopy, chromatography, multi-element analysis, and stable isotope analysis [3]. Spectroscopy and chromatography primarily conduct analyses based on organic compounds in samples, while multi-element and stable isotope analyses mainly use the elemental composition of samples for traceability purposes. However, certain organic compounds in samples are susceptible to interference from multiple factors such as growth environment and processing methods, leading to significant variability. This variability may limit the accuracy of their discriminative capabilities. By contrast, some stable isotope ratios with geographical specificity are less affected by the abovementioned interferences and can provide more reliable discriminative evidence. Among various stable isotopes, the composition of ^87^Sr/^86^Sr is closely associated with the local geological bedrock. Although the ^87^Sr/^86^Sr ratio may exhibit minor variability due to differences in bedrock weathering rates and external material inputs (e.g., fertilizer, atmospheric deposition), its isotopic fractionation effect in nature is negligible [4]. Therefore, it can serve as a robust tool for food geographical origin traceability [5,6].

After establishing a baseline map of bioavailable ^87^Sr/^86^Sr for a specific region, the statistical probability that an agricultural product originates from this region can be calculated based on the product’s ^87^Sr/^86^Sr ratios [7,8]. The direct sampling strategy refers to the direct collection of target materials for ^87^Sr/^86^Sr analysis during the construction of ^87^Sr/^86^Sr baseline maps. By directly detecting the ^87^Sr/^86^Sr signal in target materials, this method can effectively reflect the dominant geochemical characteristics of the environment in which they formed or grew, though it should be noted that mixed strontium sources (e.g., chemical fertilizers, atmospheric dust, irrigation water) may introduce minor deviations to the isotopic signal. Currently, the use of direct sampling to construct bioavailable ^87^Sr/^86^Sr baseline maps for provenance research has been widely applied in countries such as France [9], Japan [10], Denmark [11], Ireland [12], and South Korea [13].

In recent years, researchers from China have also used direct sampling to construct China’s first large-scale bioavailable Sr isotope map [14]. However, this map has limitations when applied to food origin traceability in China primarily due to its over-reliance on water samples and insufficient sample density. For China, a country with a vast geographical area and complex geological background, mapping fine-scale ^87^Sr/^86^Sr baseline maps across the entire nation in a short period is not practically feasible. This is particularly true for Eastern China, a region with abundant and diverse food resources, which urgently requires more detailed ^87^Sr/^86^Sr baseline maps to meet the demands of food origin traceability.

Located in southeastern China, Anhui Province boasts an abundance of agricultural products. Its major food crops include rice, wheat, and corn, along with a variety of medicinal and edible agricultural products such as *Chaenomeles speciosa*, *Paeonia ostii*, *Polygonatum cyrtonema*, Juhua (*Chrysanthemum morifolium*), Lingzhi (*Ganoderma lucidum*), and Fuling (*Poria cocos*). In addition, the province is renowned for producing high-quality teas, including Taiping Houkui, Huangshan Maofeng, and Lu’an Guapian. To date, Anhui Province has obtained certification for 89 geographical indication (GI) products. Leveraging the “Belt and Road” Initiative, the export volume of Anhui’s GI products grew by 20% in 2024, reaching a sales value of US$150 million. Among these, tea exports increased by 30%, and Chinese medicinal material exports rose by 25%, making Anhui a key focus area for agricultural product origin traceability research [15]. Meanwhile, Anhui Province features significant topographic relief and diverse landform types, providing ideal conditions for conducting traceability research based on ^87^Sr/^86^Sr ratios. In this study, Anhui Province was selected as the target region to construct a fine-scale bioavailable ^87^Sr/^86^Sr baseline map. This not only meets the needs of the local food traceability system but also lays a foundation for developing fine-scale Sr isotope baseline maps across China.

We developed a precise traceability tool based on bioavailable Sr to verify the authenticity and geographical origin of plant-derived foods, and systematically selected representative samples to validate the constructed ^87^Sr/^86^Sr map and analytical approach, confirming their high accuracy and reliability. The constructed ^87^Sr/^86^Sr map provides robust scientific support for protecting geographical indication products and enhancing market supervision.

## 2. Materials and Methods

### 2.1. Reagents

Hydrochloric acid (GR, Tianjin Fengchuan Chemical Reagent Co., Ltd., Tianjin, China) was purified via two rounds of sub-boiling distillation for use in resin column rinsing. Nitric acid (Trace metal grade, Fisher, Waltham, MA, USA) was used directly for sample digestion and Sr elution solution acidification. Hydrofluoric acid (Trace metal grade, Fisher, Waltham, MA, USA) enabled complete digestion of silicate samples, with residual HF removed by evaporation to prevent instrument corrosion. Perchloric acid (MOS grade, Tianjin Fengchuan Chemical Reagent Co., Ltd., Tianjin, China) oxidized organic matter and dissolved SrF_2_ precipitates to enhance Sr recovery. NBS 987 (SRM, NIST, Gaithersburg, MD, USA) is mainly used for the calibration of ^87^Sr/^86^Sr. GSB Sr (single-element Sr certified reference material, National Steel Materials Testing Center, General Institute of Steel Research, Beijing, China) is mainly used for accurate quantification of Sr element content and process quality control.

^87^Sr/^86^Sr ratios were measured using MC-ICP-MS (Neptune Plus, Thermo Fisher Scientific, Waltham, MA, USA), and elemental concentrations were determined using ICP-MS (Plasma Quant MS, German Jena Analytical Instruments Co., Ltd., Jena, Germany) during pretreatment. All experiments utilized freshly prepared Milli-Q water (18.2 MΩ·cm at 25 °C) to eliminate metal ion interference. All experiments were performed in the Class-100 clean room. Blank experiments were performed for all reagents to rule out potential interference on Sr detection.

### 2.2. Sample Collection

#### 2.2.1. Plant and Water Samples for ^87^Sr/^86^Sr Analysis

In the preliminary stage of this study, He et al. identified mixed plant samples as the optimal biological indicator for constructing the ^87^Sr/^86^Sr baseline map [16].

**Figure 1:**
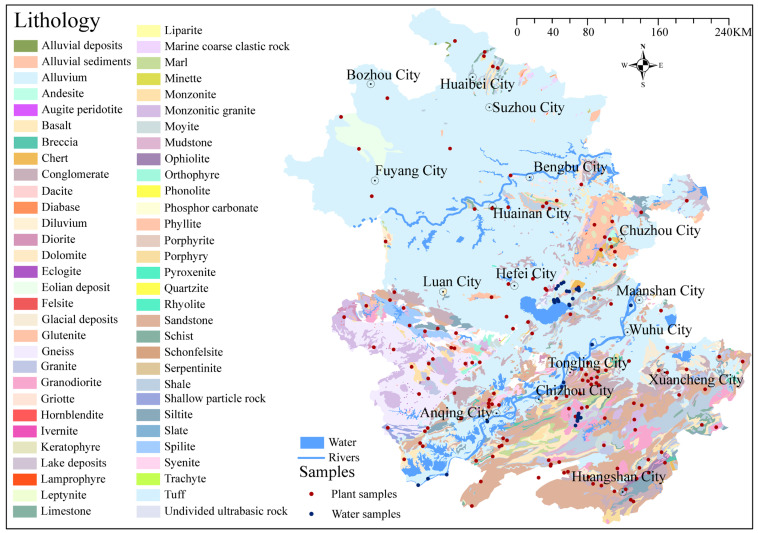
A geological map of Anhui Province with the locations of samples of plants and water.

Plant samples were collected in Anhui Province based on the sample selection method established by Willmes et al. [9], with approximately one sample collected per 900 km^2^. Willmes et al. [9] demonstrated that this sampling density is adequate for capturing fine-scale isotopic variations in geologically heterogeneous regions. Anhui Province has an area of ~140,100 km^2^ and features a complex and diverse topography, comprising mountains, hills, basins, and plains. The Yangtze and Huaihe Rivers run from west to east through the entire province. Based on the lithological characteristics of Anhui’s surface geological data at a scale of 1:200,000, published in the National Geological Archives of China (https://www.ngac.cn/125cms/c/qggnew/index.htm (accessed on 27 March 2024)), 149 sampling sites covering the main geological units of Anhui Province were selected for this study (Figure 1, Table S1). To ensure greater representativeness of the samples, sampling sites located in atypical geologic units within their geographic area (e.g., minor geologic outcrops < 10 km^2^, river terraces) were excluded. These units are not representative of the dominant geologic background of the region, and their exclusion ensures that samples primarily reflect the characteristic geochemical signals of the target study area [9]. In addition, because ^87^Sr/^86^Sr ratios are susceptible to interference from human activities, areas with frequent human disturbances (e.g., farmlands and residential areas) were excluded from this study [17].

All data for the 45 water sampling sites in Anhui Province used in this study were obtained from previously reported studies [18,19,20,21,22]. Notably, these water datasets were compiled from studies across different years with varying analytical protocols; thus, their comparability was evaluated a priori. Analytical consistency was ensured by retaining only data calibrated against NIST SRM 987 and reporting procedural blanks.

#### 2.2.2. Samples for Production Region Traceability

Two samples were used to verify the accuracy of the ^87^Sr/^86^Sr map. One was the root bark of *P. ostii*, which was collected from Fenghuangshan, Anhui Province, in 2015. The other was the fruit of *Ch. speciosa*, which was collected in 2024 from Xuanzhou District, Anhui Province. Both samples were provided by Dr. Zhao Yujiao, School of Pharmacy, Anhui University of Chinese Medicine. In addition, to further improve the reference system for origin traceability research on teas and medicinal and edible products in Anhui Province, we retrieved and cited partial GI products, GI certification trademarks, and GI agricultural products of Anhui Province from the Protected Geographical Indication (http://www.cpgi.org.cn (accessed on 12 September 2025)) (Table S3).

### 2.3. ^87^Sr/^86^Sr Determination

#### 2.3.1. Specimen Preprocessing Procedures

First, 2 g of the plant sample was weighed and ultrasonically cleaned three times with Milli-Q water. After vacuum drying, the sample was ashed at 600 °C for 6 h. The dehydrated plant sample was then pulverized and homogenized through a 200-mesh sieve (aperture: 0.075 mm), awaiting subsequent fractionation and purification processes.

#### 2.3.2. Sample Digestion

200 mg of the ashed plant powder was transferred into a polytetrafluoroethylene digestion vessel. A total of 3 mL concentrated HNO_3_ was added, and the mixture was allowed to soak overnight before being evaporated to dryness. Next, aqua regia and a small amount of HF were added to immerse the sample, followed by sealed heating on a hot plate at 70–80 °C for 12 h. If significant bubbling occurred, the sample was evaporated to dryness, and the aforementioned heating step was repeated. If undissolved residues remained, an appropriate amount of HClO_4_ was added, and the mixture was heated at 150 °C for another 12 h [23].

Subsequently, 1 mL of 3.5 mol/L HNO_3_ was added to the evaporated plant samples, which were kept warm for 1 h to ensure complete dissolution of the samples in HNO_3_ for subsequent analysis. The Sr concentration in the solutions was determined using ICP-MS. Based on the measured concentrations, the volume of solution required for column separation was calculated [24].

#### 2.3.3. Sr/Matrix Separation

For Sr isotope separation, Sr-specific resin (Triskem, Bruz, France, 100–150 μm, 0.5 mL column bed volume) was used. First, 7 mL of 3.5 mol/L HNO_3_ was used to elute major and rare earth elements. Next, the Sr Spec resin column was rinsed with 4 mL of Milli-Q water, and the eluate containing Sr was collected. A constant-flow pump was used to control the flow rate (0.5–1.0 mL/min) throughout the process, ensuring sufficient interaction between target ions and the resin to avoid incomplete separation. After the purified Sr was evaporated to dryness, 1 mL of 2% HNO_3_ was added to reconstitute the sample, which was then ready for ICP-MS analysis. The recovery efficiency of Sr using this protocol was verified with the certified reference material NIST SRM 1515, yielding a recovery of 90%, which meets the sample purity and yield requirements for ^87^Sr/^86^Sr isotope ratio analysis.

#### 2.3.4. ICP-MS and MC-ICP-MS Measurements

To ensure the accuracy of the results, duplicate samples were included in each batch of sample testing. The measurement of ^83^Kr was conducted to monitor the interference of ^84^Kr and ^86^Kr on ^84^Sr and ^86^Sr, and the measurement of ^85^Rb was performed to monitor the interference of ^87^Rb on ^87^Sr. The instrumental fractionation of Sr isotopes was corrected using an exponential equation, with calibration based on the ^88^Sr/^86^Sr ratio = 8.375209 [25].

The measured ^87^Sr/^86^Sr ratio of NBS 987 in this study was 0.7102478 ± 0.000017 (2σ). Analytical precision is expressed as 2σ values, derived from 60 replicate measurements of the NBS 987 to represent long-term instrumental stability.

Full-process quality control (QC) samples and procedural blanks were analyzed every 10 samples. The QC samples were laboratory control samples (LCSs), prepared by spiking a blank matrix with a known amount of Sr standard, and used to verify batch-to-batch consistency. Procedural blanks were employed to correct for potential Sr contamination from reagents, laboratory ware, or the analytical process. Prior to sample analysis, the MC-ICP-MS system was optimized using a 200 μg/L Sr standard solution. Plasma interface parameters and ion lens voltages were adjusted to maximize Sr ion sensitivity, ensuring reliable detection of target isotopes.

### 2.4. Creation of the Bioavailable ^87^Sr/^86^Sr Map

First, Origin 2024 software was used to analyze the ^87^Sr/^86^Sr measurement data from 149 plant samples, with outliers removed using the interquartile range (IQR) method. ESRI ArcGIS 10.8 was used for spatial analysis and predictive mapping. The ^87^Sr/^86^Sr ratio data were spatially integrated with the 1:200,000 surficial geological data of Anhui Province. Ordinary Kriging interpolation was used to generate the ^87^Sr/^86^Sr baseline map of Anhui Province, and the quality of the map was evaluated through cross-validation. To test the predictive power of the model, we calculated the root mean square error (RMSE), mean absolute error (MAE) and residuals of the model to estimate the spatial uncertainty.

## 3. Results

### 3.1. Construction of a Bioavailable ^87^Sr/^86^Sr Map for Anhui Province

The ^87^Sr/^86^Sr ratios of all plant samples ranged from 0.7073 to 0.7216, with a mean value of 0.7114 ± 0.0021 (mean ± SD, n = 149) (Table S1). Repeated measurements of the NBS 987 standard reference material yielded an ^87^Sr/^86^Sr ratio of 0.710248 ± 0.000017 (2SD, n = 7), which is consistent with the recommended ^87^Sr/^86^Sr ratio of 0.710248 for NBS 987 [26]. This confirms the reliability of the analytical results. For water samples from Anhui Province, the ^87^Sr/^86^Sr ratios ranged from 0.7094 to 0.7144, with a mean value of 0.7110 ± 0.001 (mean ± SD, n = 45) (Table S2). Notably, the ^87^Sr/^86^Sr ratios of plant samples exhibit a wider range than those of water samples. This difference arises because water bodies undergo extensive mixing via hydrological processes, which homogenizes their ^87^Sr/^86^Sr signatures and reduces spatial isotopic variability. In contrast, plants integrate and retain the ^87^Sr/^86^Sr signal of their immediate growing environment without significant homogenization. Therefore, constructing a ^87^Sr/^86^Sr baseline map using plant samples has the potential to better capture the fine-scale spatial variability of ^87^Sr/^86^Sr ratios in the study area.

Anhui Province administers 16 prefecture-level cities, among which Xuancheng, Huangshan, and Anqing exhibit relatively complex lithology. The average bioavailable ^87^Sr/^86^Sr ratios of plants in these regions are presented in Table 1 As a mountainous region, Huangshan City has bedrock rich in conglomerate, granodiorite, ophiolite, and shale, and the highest average ^87^Sr/^86^Sr ratio of plants in this region is 0.7143 ± 0.003 (n = 21). As a hilly region, Tongling City has bedrock rich in sandstone and alluvial deposits, and the lowest average ^87^Sr/^86^Sr ratio of plants in this region is 0.7091 ± 0.001 (n = 8). As a plain region, Fuyang City has bedrock rich in alluvial deposits, and the average ^87^Sr/^86^Sr ratio of plants in this region is 0.7118 ± 0.0002 (n = 4). Integrating the average ^87^Sr/^86^Sr ratios with lithological variations enables intuitive visualization of the relationship between lithology and the ^87^Sr/^86^Sr composition of plants.

**Figure 2:**
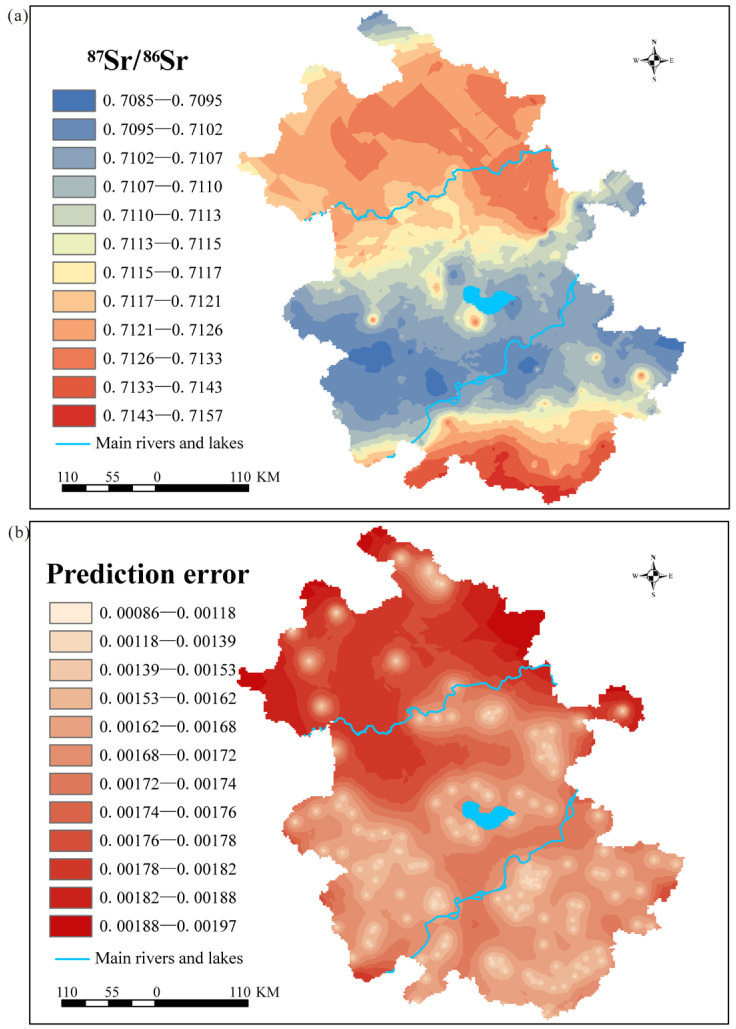
^87^Sr/^86^Sr baseline for Anhui Province. (**a**) Predicted Sr isotope ratios for Anhui Province based on kriging interpolation; (**b**) Residual analysis using cross-validation methods.

Based on the above results, this study first removed 6 outliers, which were outside the 1.5 IQR, from the 149 plant ^87^Sr/^86^Sr data using the IQR method. The remaining 143 plant ^87^Sr/^86^Sr ratios exhibited a normal distribution. Subsequently, an ^87^Sr/^86^Sr baseline map was generated using the ordinary Kriging interpolation method (Figure 2a, Table 2). Afterwards, cross-validation was conducted to evaluate the spatial uncertainty of the kriging interpolation (Figure 2b). The kriging interpolation results in an MAE of 0.00164 (11.5% of the whole Sr isotope dataset range) and an RMSE of 0.00166 (11.6% of the whole Sr isotope dataset range), suggesting a good fit between the observed and predicted ratios.

Figure 2b shows a small prediction error with a regional error range of 0.00086–0.00197. This indicates that the ^87^Sr/^86^Sr data in most areas of Anhui Province are highly consistent with the baseline predictions, and the baseline model is effective for the spatial prediction of regional ^87^Sr/^86^Sr ratios. In the Huaibei Plain region, the sample dispersion is slightly higher owing to the presence of thick loess deposits. This corresponds to slightly larger local fluctuations in the error map; however, the overall values remain within a reasonable range.

This study presents the first detailed baseline map of bioavailable ^87^Sr/^86^Sr for Anhui Province. Figure 2a shows that the regional ^87^Sr/^86^Sr ratios in northern and southern Anhui Province are higher than those in central, southwestern, and southeastern Anhui. Across the entire province, ^87^Sr/^86^Sr ratios exhibit a trend of higher values in the north and south and lower values in the central region, with significant regional differences in this map attributed to bedrock influence. These patterns reflect local heterogeneous geological characteristics and demonstrate considerable variability.

### 3.2. Application of Anhui Province’s ^87^Sr/^86^Sr Map in Food Geographical Origin Traceability

#### 3.2.1. *Paeonia ostii*

*P. ostii* is a perennial deciduous shrub belonging to the Paeoniaceae family. It is celebrated for its large, ornamental flowers. These blooms are not only valued as a famous ornamental plant but also used in the production of pastries and beverages. The kernel of *P. ostii* contains ~30% oil, which is rich in unsaturated fatty acids such as α-linolenic acid, linoleic acid, and oleic acid. The total content of these unsaturated fatty acids reaches up to 90% [27]. This unique fatty acid endows *P. ostii* seed oil with multiple therapeutic effects, including antithrombotic, anti-inflammatory, and antitumor effects, as well as the ability to enhance immunity. As a result, *P. ostii* seed oil is classified as a high-end woody edible oil with exceptional nutritional and health-promoting values [28,29].

Tongling and Bozhou in Anhui Province are renowned production regions for *P. ostii*. In traditional Chinese medicine, the root bark of *P. ostii* is commonly used to treat diseases. It is recognized that the Dao-di *P. ostii* is produced in Fenghuangshan, located at the junction of Yian District (Tongling City) and Nanling County (Wuhu City) in Anhui Province, and it is commonly known as “Fengdanpi.” *P. ostii* grown in this area exhibits superior quality. However, it requires a growth cycle of >5 years and yields relatively low output. In the 1960s and 1970s, *P. ostii* was introduced to Bozhou, Anhui Province, and the resulting product is known as “Bodanpi.” Bozhou-grown *P. ostii* has a shorter cultivation period (only 4 years) and higher yield, currently accounting for ~50% of China’s total *P. ostii* output. Owing to its superior efficacy, low yield, and high popularity among consumers, Fengdanpi is typically priced higher than Bodanpi. Therefore, it is highly necessary to distinguish the geographical origins of these two products.

**Figure 3:**
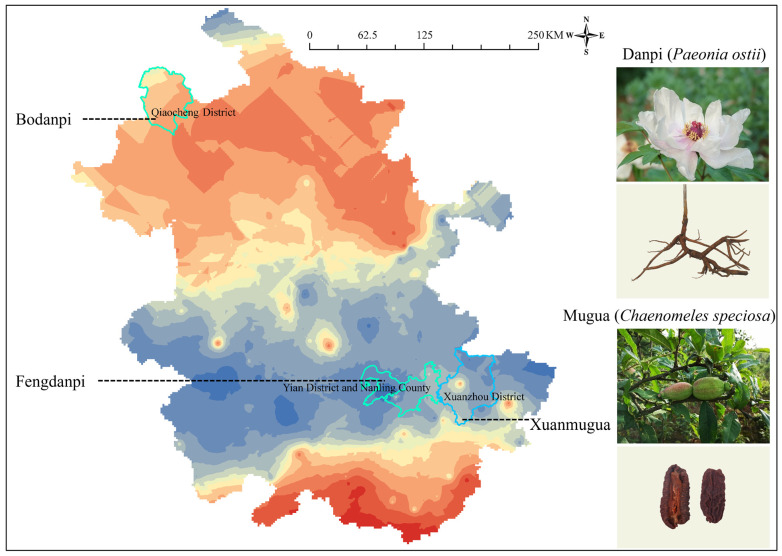
Distribution of ^87^Sr/^86^Sr in the *Paeonia ostii* and *Chaenomeles speciosa* of Anhui Province.

To verify whether this baseline map can effectively distinguish between the production regions of Fengdanpi and Bodanpi, a sample of Fengdanpi provided by Anhui University of Chinese Medicine was analyzed. The ^87^Sr/^86^Sr ratio of this Fengdanpi, originating from Fenghuangshan, was 0.7091. As shown in Figure 3, Yi’an District and Nanling County, the geographical origins of this Fengdanpi, are dominated by alluvial rocks and sandstones, with a local ^87^Sr/^86^Sr ratio range of 0.7085–0.7113. By contrast, Qiaocheng District, the main production area of Bodanpi, has a geological background primarily comprising alluvial rocks, and its ^87^Sr/^86^Sr ratio ranges from 0.7115 to 0.7126. Notably, there is a distinct difference in the ^87^Sr/^86^Sr ratio intervals between the two production regions.

When this measured ^87^Sr/^86^Sr ratio was compared against the Anhui Province ^87^Sr/^86^Sr baseline map constructed in this study, it was found to fall exactly within the ratio range of 0.7085–0.7113 corresponding to Yi’an District and Nanling County. This result is entirely consistent with the origin-tracing conclusion based on the ^87^Sr/^86^Sr ratio division of production regions. These findings confirm that the ^87^Sr/^86^Sr ratio can serve as an effective indicator for distinguishing the geographical origins of Fengdanpi and Bodanpi.

#### 3.2.2. *Chaenomeles speciosa*

*Ch. speciosa* is a perennial deciduous shrub belonging to the Rosaceae family. Its fruits emit a rich aroma and are abundant in various organic acids (e.g., oleanolic acid and ursolic acid) and vitamins. In addition to being consumed fresh, the fruits are widely used in processing, including the production of preserved fruits, fruit juices, and fruit wine. Traditionally, the “Xuanmugua” produced in Xuanzhou District, Anhui Province, is a Dao-di herb and a premium edible product, renowned for its superior quality.

To verify the applicability of this baseline map for origin traceability, the ^87^Sr/^86^Sr ratio of a Xuanmugua sample, provided by Anhui University of Chinese Medicine, was determined, yielding a measured value of 0.7121. Based on the Anhui Province ^87^Sr/^86^Sr baseline map constructed in this study (Figure 3), Xuanzhou District is dominated by three main geological types, namely, conglomerates, glacial deposits, and sedimentary rocks, with a corresponding ^87^Sr/^86^Sr ratio range of 0.7107–0.7126. When this measured ratio was spatially compared against the aforementioned baseline map, it fell exactly within the ^87^Sr/^86^Sr interval corresponding to Xuanzhou District. This result is highly consistent with the division of production regions based on geological backgrounds, confirming that the sample is indeed of Xuanzhou District origin. It further validates the feasibility and accuracy of using the ^87^Sr/^86^Sr baseline map for *Ch. speciosa* geographical origin traceability.

#### 3.2.3. Teas

China is the origin of tea. The region south of the Huaihe River in Anhui Province is a key production area for premium tea in China, yielding a variety of high-quality tea types, such as Huangshan Maofeng, Qimen Hongcha, Lu’an Guapian, Huoshan Huangya, Tongcheng Xiaohua, and Taiping Houkui. These teas not only enjoy a prestigious reputation in China but are also sold worldwide.

The tea industry in Anhui Province boasts a substantial scale. For instance, the cultivation area of “Huangshan Maofeng” spans ~366.67 km^2^, with an annual output of 15,000 tons of finished tea and a comprehensive annual output value of approximately US$1.817 billion. For “Lu’an Guapian”, the total tea plantation area is ~133.33 km^2^, the annual output of finished tea reaches 11,800 tons, and the direct annual output value is approximately US$244 million [30].

**Figure 4:**
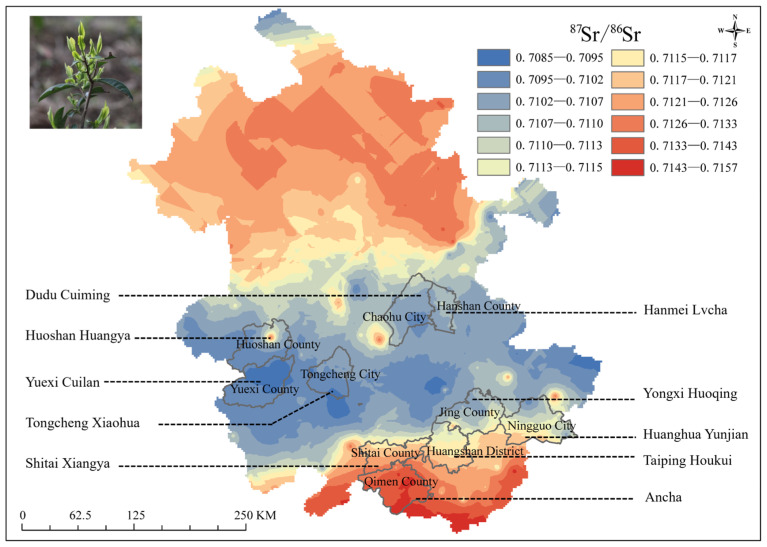
Distribution of ^87^Sr/^86^Sr in major tea-producing regions of Anhui Province.

The ^87^Sr/^86^Sr map of Anhui Province constructed in this study provides a robust basis for tracing the geographical origins of Anhui-grown teas. As illustrated in Figure 4, teas such as An cha, Shitai Xiangya, Taiping Houkui, Huanghua Yunjian, and Yongxi Huoqing are produced in the mountains in southern Anhui. The geological background of this region is dominated by granitoids, low-grade metamorphic rocks, and sedimentary rocks, with a primary ^87^Sr/^86^Sr ratio range of 0.7115–0.7157. Teas including Yuexi Cuilan and Huoshan Huangya originate from the Dabie Mountains in western Anhui, which mainly comprise medium- to high-grade metamorphic rocks. Their corresponding ^87^Sr/^86^Sr ratio ranges from 0.7085 to 0.7107. Teas such as Tongcheng Xiaohua, Hanmei Lvcha, and Dudu Cuiming are produced in the Jianghuai Hills. Dominated by sedimentary rocks, this region has a primary ^87^Sr/^86^Sr ratio range of 0.7107–0.7113. These data clearly demonstrate that there are significant differences in ^87^Sr/^86^Sr ratios among different tea-producing regions. This unique “geological fingerprint” can serve as a key indicator for identifying the geographical origins of teas.

#### 3.2.4. *Platycodon grandiflorum*

**Figure 5:**
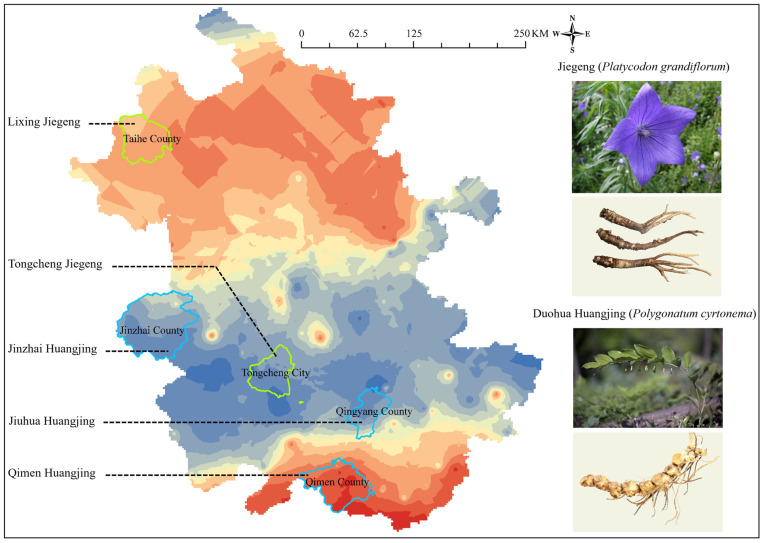
Distribution of ^87^Sr/^86^Sr in the *Platycodon grandiflorum* and *Polygonatum cyrtonema* of Anhui Province.

*P. grandiflorum* is a medicinal and edible plant whose dried root is used for various purposes [31]. It is rich in diverse nutritional components, including essential amino acids, proteins, dietary fiber, vitamins, and unsaturated fatty acids. In addition, it contains bioactive compounds such as *P. grandiflorum* saponins, flavonoids, polyalkyne, fatty acids, sterols, and phenolic acids. These components confer multiple pharmacological effects, including expectorant, antitussive, anti-inflammatory, anti-obesity, hypoglycemic, hypolipidemic, antitumor, and hepatoprotective activities, making *P. grandiflorum* highly valuable in the pharmaceutical, healthcare, and food industries [32]. Anhui Province is a major production area of *P. grandiflorum*. Beyond its medicinal use, Anhui-sourced *P. grandiflorum* is also developed into food products (e.g., Jiegeng pickles and Jiegeng wine) and exported in large quantities to countries such as South Korea. Currently, two Jiegeng varieties from Anhui have obtained national GI certification, namely, Lixing Jiegeng and Tongcheng Jiegeng. Lixing Jiegeng is produced in Taihe County, where the geological formations are characterized by a predominance of alluvial and eolian deposits. The ^87^Sr/^86^Sr ratio range in this region is 0.7117–0.7126. Tongcheng Jiegeng originates from Tongcheng City and is characterized by geological formations of diorite, syenite porphyry, and gneiss. The corresponding ^87^Sr/^86^Sr ratio range here is 0.7085–0.7113 (Figure 5). Notably, there is a distinct difference in the ^87^Sr/^86^Sr ratio intervals between the two GI Jiegeng varieties.

#### 3.2.5. *Polygonatum cyrtonema*

*P. cyrtonema* is a known medicinal plant, and its roots are recorded in the *China Pharmacopoeia* and have been used as food and medicine since ancient times [33]. Studies have demonstrated that *P. cyrtonema* exhibits pharmacological effects such as hypoglycemia and hypolipidemia activities and is widely used in the intensive processing of food products and health supplements [34,35]. To meet market demand, large-scale cultivation of *P. cyrtonema* has been established in Anhui Province. The main cultivation areas include Jinzhai County in the Dabie Mountains region, as well as Qingyang County and Qimen County in the mountains in southern Anhui.

Currently, the varieties of Huangjing in Anhui Province that have obtained National Geographical Indication certification include Jinzhai Huangjing, Jiuhua Huangjing, and Qimen Huangjing. In terms of geochemical characteristics (Figure 5), the geological background of the Jinzhai County region is dominated by granitoids, gneiss, syenite, conglomerate, and sandstone, with an ^87^Sr/^86^Sr ratio ranging from 0.7095 to 0.7113. The Qingyang County region, the producing area of Jiuhua Huangjing, has a geological background mainly comprising granitoids, conglomerate, and sandstone. Its ^87^Sr/^86^Sr ratio ranges from 0.7085 to 0.7107. The Qimen County area, where Qimen Huangjing grows, has a geology characterized by conglomerate, sandstone, shale, and granodiorite, with an ^87^Sr/^86^Sr ratio that ranges from 0.7126 to 0.7157. Jinzhai County and Qingyang County can be distinguished from Qimen County. However, additional methods are required to differentiate between Jinzhai County and Qingyang County themselves.

## 4. Discussion

### 4.1. Construction of the First Fine-Scale ^87^Sr/^86^Sr Map of Anhui Province

Herein, the first fine-scale ^87^Sr/^86^Sr map of Anhui Province was successfully constructed. This map provides critical regional high-resolution data for global ^87^Sr/^86^Sr geochemical mapping. From a global perspective, ^87^Sr/^86^Sr mapping has achieved significant progress in regions such as the United States, Australia, and continental Europe [36,37,38]. By establishing basic spatial databases of ^87^Sr/^86^Sr ratios in surface environments, these countries have provided important reference tools for archeological migration studies and food origin traceability. Alternatively, despite China’s vast territory and complex, diverse geological settings, ^87^Sr/^86^Sr mapping at the national scale is still in the preliminary development stage. Anhui Province covers an area of ~140,000 km^2^. As a geologically representative province in Eastern China, it has an area equivalent to 39% of Germany’s land area. Achieving high-precision mapping in this region is not only a crucial supplement to the global ^87^Sr/^86^Sr database but also a key step in promoting the systematization and in-depth development of research in this field in China.

The first bioavailable ^87^Sr/^86^Sr baseline map of Anhui Province constructed in this study features high resolution and high precision, providing a solid data foundation for origin traceability research on plant-derived products within the region. Compared with published large-scale Chinese ^87^Sr/^86^Sr baseline maps, which exhibit a higher RMSE of 0.00168 and a lower sampling density (2 samples per 10,000 km^2^), the Anhui ^87^Sr/^86^Sr map offers distinct advantages in complex landforms (e.g., mountains and hills). The large-scale map struggles to capture subtle regional isotopic variations owing to resolution constraints [14], whereas the baseline map in this study more accurately reflects actual ^87^Sr/^86^Sr variations in such regions.

Taking Anqing City, an area with complex landforms, as an example, the ^87^Sr/^86^Sr ratio of this region in Anhui’s ^87^Sr/^86^Sr map is significantly lower than that of the corresponding region in China’s national ^87^Sr/^86^Sr map. Beyond the influence of Archean–Lower Proterozoic granites, this difference is more closely associated with the extensive outcrops of Paleozoic–Early Mesozoic marine carbonate rocks, clastic rocks, and partial mafic rocks in the area; i.e., marine carbonate rocks are prone to weathering and exhibit relatively low ^87^Sr/^86^Sr ratios (~0.707–0.710), which directly results in the relatively low ^87^Sr/^86^Sr ratio (~0.7073) of local plants. In addition, because of the extensive distribution of sandstone and granodiorite in the mountains in southern Anhui, the ^87^Sr/^86^Sr ratios of local plants are relatively high. By contrast, the central Anhui area, characterized by widespread carbonate and clastic rocks, exhibits generally low ^87^Sr/^86^Sr ratios in local plants. These correlations, where regional lithologic variations directly align with measurable differences in plant ^87^Sr/^86^Sr ratios, validate that these ratios accurately reflect the isotopic signatures of the plants’ growth environments and provide critical evidence for the precise geographical traceability of plant-based foods.

Compared with the ^87^Sr/^86^Sr maps constructed based on water samples in previous studies [13,39], the plant samples selected in this study not only cover a wider sampling range but also more directly reflect the isotopic characteristics of bioavailable Sr. ^87^Sr/^86^Sr maps constructed using plant samples exhibit higher data reliability and applicability in the traceability of plant-derived foods. To further enhance regional representativeness, this study used mixed herbaceous plant samples to characterize the regional average bioavailable ^87^Sr/^86^Sr ratio. This approach effectively avoids potential random biases associated with single species or individual samples, thereby improving the reliability of the data [16].

### 4.2. Feasibility Analysis of Tracing Plant-Derived Food Production Areas Using the ^87^Sr/^86^Sr Map of Anhui Province

Anhui Province features diverse topography and landforms, encompassing geographical units such as the Dabie Mountains area, mountains in southern Anhui, Jianghuai Hills, plains and hills along the Yangtze River, and Huaibei Plain. Climatically, it spans the southern temperate zone, northern subtropical zone, and middle subtropical zone. This region not only cultivates food crops over large areas but also extensively grows various medicinal plants. To date, the number of national GI products in Anhui Province has increased to 181, occupying an important position nationwide. The construction of a fine-scale ^87^Sr/^86^Sr map provides a scientific basis for product traceability based on geological background differences.

Currently, ^87^Sr/^86^Sr has been widely applied in origin traceability research for specialty agricultural products, such as Tiepi Shihu (*Dendrobium officinale*), Huangjing (*Polygonatum* spp.), and wine [40,41,42]. This technique can effectively combat counterfeit products and protect consumers’ rights and interests. In previous studies, ^87^Sr/^86^Sr has often been used as a single variable in traceability models, which fails to fully leverage the specific tracing capability of ^87^Sr/^86^Sr. To a certain extent, this limitation has restricted the accuracy and reliability of traceability results. By contrast, constructing a bioavailable ^87^Sr/^86^Sr baseline map for a specific region enables a more precise capture of the isotopic correlation between the production environment and target products, thereby maximizing the core advantages of ^87^Sr/^86^Sr in tracing. Meanwhile, from a long-term development perspective, the systematic construction of regional ^87^Sr/^86^Sr maps not only reduces the cost of investment for individual traceability analyses but also provides stable foundational data support for the continuous conduct of multicategory and cross-scale origin traceability research. Thus, it represents an economical and sustainable approach to traceability.

Based on the bioavailable ^87^Sr/^86^Sr baseline map of Anhui Province constructed in this study, origin identification of tea varieties from different geographical sources in Anhui Province can be achieved. Thus, the ^87^Sr/^86^Sr baseline map can serve as a key isotopic indicator for distinguishing tea origins. Further application of the Anhui ^87^Sr/^86^Sr map to origin traceability verification of medicinal and edible agricultural products showed that distinct differences also existed in the ^87^Sr/^86^Sr ratio ranges of specific regions. These regions include Jinzhai County, Qingyang County, and Qimen County, which produce *P. sibiricum*, and Taihe County and Tongcheng County, which produce *P. grandiflorum*. This method is, therefore, also applicable for distinguishing the geographical origins of medicinal and edible agricultural products in Anhui Province. In addition, using the bioavailable ^87^Sr/^86^Sr baseline map of Anhui Province, this study could accurately locate the production area of Fengdanpi to the Fenghuangshan area of Anhui Province and trace the origin of *C. speciosa* to Xuanzhou District. These results are highly consistent with the recorded traditional Dao-di producing areas, confirming the effectiveness of the ^87^Sr/^86^Sr map constructed in this study for practical origin traceability applications.

In conclusion, the bioavailable ^87^Sr/^86^Sr baseline map of Anhui Province provides a novel approach for tracing the geographical origin of leafy, fruity, and root-based agricultural products. In addition, it offers an important reference for low-resolution maps to narrow the range of potential production areas. We verified the effectiveness of this high-resolution ^87^Sr/^86^Sr map. It can not only meet the origin-tracing requirements of different plant organ types but also cope with the challenges of origin-tracing under complex geological conditions. This map offers robust scientific support for food quality supervision and GI protection while also contributing detailed plant-derived data to the development of a more comprehensive national Sr isotope map of China. Overall, this baseline map holds broad application prospects in future food traceability and related fields. However, future work should take three key limitations into consideration: (1) Validation scope: The current traceability validation focuses on five foods, and further verification is needed for other agricultural products (e.g., rice) with different Sr uptake characteristics. (2) External factor impacts: The potential effects of long-term excessive chemical fertilization or industrial dust deposition on plant Sr isotope ratios were not systematically evaluated, which may affect traceability accuracy in specific polluted areas. (3) Temporal variability: The baseline map is based on samples collected during 2023–2024, and the stability of bioavailable Sr isotopes over seasonal and interannual scales requires long-term monitoring.

## 5. Conclusions

By analyzing the ^87^Sr/^86^Sr values of 149 plant samples collected from Anhui Province, this study successfully constructed the first fine-scale bioavailable ^87^Sr/^86^Sr baseline map that meets the requirements for tracing the origin of plant-derived food-producing areas in Anhui Province. This study revealed significant spatial variability in the ^87^Sr/^86^Sr ratios of plants across Anhui Province. In particular, plants in the mountains in southern Anhui, where sandstone and granodiorite rocks are widely distributed, and those in the Huaibei Plain, affected by thick loess deposits, exhibited relatively high ^87^Sr/^86^Sr values. By contrast, plants in the hills in central Anhui generally had lower ^87^Sr/^86^Sr ratios, attributed to the region’s high abundance of carbonate rocks and clastic rocks. This spatial variation is consistent with the diverse lithological characteristics of Anhui Province, validating previous expectations that ^87^Sr/^86^Sr ratios in this region are highly variable. Furthermore, this study addresses the limitations of existing large-scale baseline maps, which lack sufficient representativeness in areas with complex lithology.

In this study, the ^87^Sr/^86^Sr baseline map was successfully applied to trace the origins of teas, *Ch. speciosa*, *P. ostii*, *P. grandiflorus*, and *P. sibiricum*. This application validated the effectiveness of the baseline map in origin-tracing research for leafy, fruity, and root-based agricultural products. The map not only provides a reliable tool for identifying food-producing areas in Anhui Province but also offers robust support and methodological reference for quality control, protection, and market supervision of GI products.

Furthermore, with the supplementary measurement of additional plant samples, the ^87^Sr/^86^Sr reference databases for both Anhui Province and China can be further refined and updated. This will lay a solid foundation for constructing a more accurate and comprehensive national ^87^Sr/^86^Sr baseline map of China, thereby continuously advancing the development of food traceability and related fields.

## Figures and Tables

**Table 1:** The mean ^87^Sr/^86^Sr of varies region in Anhui Province.

Location	Main Bedrock	Latitude and Longitude	Minimum ^87^Sr/^86^Sr	Maximum ^87^Sr/^86^Sr	Mean ^87^Sr/^86^Sr ± SD (n)
Hefei City	Alluvium, Conglomerate, Porphyrite	31.861 N, 117.282 E	0.7081	0.7146	0.7106 ± 0.002 (n = 13)
Chuzhou City	Phyllite, Shale	32.247 N, 118.340 E	0.7084	0.7184	0.7123 ± 0.003 (n = 15)
Chizhou City	Dolomite, Limestone, Sandstone	29.693 N, 117.568 E	0.7094	0.7176	0.7117 ± 0.002 (n = 15)
Xuancheng City	Alluvium, Dolomite, Mudstone, Sandstone	30.932 N, 118.756 E	0.7087	0.7145	0.7110 ± 0.002 (n = 17)
Huangshan City	Conglomerate, Granodiorite, Ophiolite, Sandstone, Shale	30.298 N, 118.142 E	0.7101	0.7216	0.7143 ± 0.003 (n = 21)
Lu’an City	Conglomerate, Gneiss, Sandstone	31.728 N, 116.488 E	0.7091	0.7149	0.7107 ± 0.002 (n = 15)
Anqing City	Alluvium, Diorite, Gneiss, Granite, Monzonite, Sandstone	30.506 N, 117.038 E	0.7073	0.7120	0.7096 ± 0.001 (n = 22)
Tongling City	Alluvium, Sandstone	30.818 N, 117.764 E	0.7083	0.7102	0.7091 ± 0.001 (n = 8)
Maanshan City	Alluvium	31.706 N, 118.526 E	0.7102	0.7118	0.7111 ± 0.0007 (n = 3)
Wuhu City	Sandstone	31.361 N, 118.434 E	0.7086	0.7132	0.7106 ± 0.002 (n = 5)
Fuyang City	Alluvium	32.869 N, 115.861 E	0.7116	0.7122	0.7118 ± 0.0002 (n = 4)
Huainan City	Limestone	32.133 N, 116.542 E	0.7112	0.7127	0.7119 ± 0.0006 (n = 3)
Suzhou City	Shale	34.053 N, 117.222 E	0.7114	0.7137	0.7124 ± 0.0008 (n = 5)
Bozhou City	Alluvium	33.863 N, 115.734 E	—	—	0.7124 ± 0.0000 (n = 2)
Bengbu City	Gneiss	32.924 N, 117.363 E	—	—	0.7106 ± 0.0000 (n = 1)

**Table 2:** Kriging method parameters.

Method	Transformation	Trend Removal	Variogram Model	Search Neighborhood	Sectors	RMSE	MAE
Ordinary kriging	Box–Cox	3	Exponential	Standard Min: 2 Max: 5	4	0.00166	0.00164

## Data Availability

Data are contained within the article. Further information can be obtained by contacting the corresponding author.

## Supplementary Materials

The following supporting information can be downloaded at: https://www.mdpi.com/article/10.3390/foods15010033/s1, Table S1: Sample information of plants. Table S2: Sample information of water. Table S3: Anhui Province geographical indication products, trademarks and agricultural products used for traceability cases.

## Abbreviations

The following abbreviations are used in this manuscript:

GI	Geographical indication
IQR	Interquartile range
RMSE	Root mean square error
