# Supplementary material for: Fine-Scale Strontium Isotope Mapping in Eastern China (Anhui Province) and Its Application in Food Traceability
Source: Foods. 2025 Dec 22;15(1):33. doi: 10.3390/foods15010033 (PMC12785725; doi:10.3390/foods15010033)
Supplement: Supplementary file 1 [file foods-15-00033-s001.zip › Supporting Information.pdf]

**Supplementary materials**

**Supplementary Table S1** Sample information of plants.

| Sample Number | Lithology | Longitude | Latitude | $^{87}Sr/^{86}Sr$ | 2σ |
|---|---|---|---|---|---|
| AH-1 | Alluvium | 118.723 E | 31.570 N | 0.7102 | 0.000011 |
| AH-2 | Shale | 118.042 E | 31.700 N | 0.7118 | 0.000010 |
| AH-3 | Alluvium | 118.214 E | 31.637 N | 0.7112 | 0.000011 |
| AH-4 | Conglomerate | 117.802 E | 31.533 N | 0.7081 | 0.000010 |
| AH-5 | Chert | 117.882 E | 31.808 N | 0.7097 | 0.000013 |
| AH-6 | Alluvium | 117.419 E | 31.893 N | 0.7112 | 0.000010 |
| AH-7 | Alluvium | 117.419 E | 31.893 N | 0.7114 | 0.000013 |
| AH-8 | Phosphor carbonate | 117.559 E | 31.779 N | 0.7093 | 0.000011 |
| AH-9 | Gneiss | 117.546 E | 31.793 N | 0.7119 | 0.000010 |
| AH-10 | Glutenite | 116.998 E | 31.706 N | 0.7131 | 0.000010 |
| AH-11 | Shale | 117.411 E | 31.337 N | 0.7146 | 0.000012 |
| AH-12 | Liparite | 117.217 E | 31.385 N | 0.7118 | 0.000012 |
| AH-13 | Conglomerate | 117.370 E | 31.453 N | 0.7119 | 0.000012 |
| AH-14 | Porphyrite | 117.170 E | 31.840 N | 0.7083 | 0.000010 |
| AH-15 | Porphyrite | 117.170 E | 31.840 N | 0.7085 | 0.000012 |
| AH-16 | Porphyrite | 117.171 E | 31.841 N | 0.7085 | 0.000011 |
| AH-17 | Shale | 117.590 E | 32.607 N | 0.7130 | 0.000011 |
| AH-18 | Shale | 117.520 E | 32.629 N | 0.7121 | 0.000012 |
| AH-19 | Phyllite | 117.557 E | 32.665 N | 0.7122 | 0.000012 |
| AH-20 | Griotte | 117.661 E | 32.689 N | 0.7139 | 0.000012 |
| AH-21 | Siltite | 118.522 E | 32.570 N | 0.7084 | 0.000012 |
| AH-22 | Phyllite | 118.227 E | 32.477 N | 0.7138 | 0.000010 |
| AH-23 | Porphyrite | 117.912 E | 32.854 N | 0.7184 | 0.000011 |
| AH-24 | Phyllite | 118.046 E | 32.443 N | 0.7143 | 0.000011 |
| AH-25 | Mudstone | 118.151 E | 32.319 N | 0.7157 | 0.000011 |

| | | | | | |
|---|---|---|---|---|---|
| AH-26 | Andesite | 118.202 E | 32.295 N | 0.7090 | 0.000012 |
| AH-27 | Limestone | 118.219 E | 32.218 N | 0.7102 | 0.000013 |
| AH-28 | Chert | 118.113 E | 32.189 N | 0.7097 | 0.000012 |
| AH-29 | Alluvial sediments | 118.254 E | 32.170 N | 0.7113 | 0.000010 |
| AH-30 | Alluvium | 118.253 E | 32.034 N | 0.7123 | 0.000010 |
| AH-31 | Lake deposits | 118.985 E | 32.688 N | 0.7108 | 0.000011 |
| AH-32 | Sandstone | 116.798 E | 29.583 N | 0.7135 | 0.000012 |
| AH-33 | Shale | 117.117 E | 30.272 N | 0.7142 | 0.000012 |
| AH-34 | Sandstone | 116.889 E | 29.832 N | 0.7147 | 0.000012 |
| AH-35 | Sandstone | 117.155 E | 30.251 N | 0.7176 | 0.000011 |
| AH-36 | Limestone | 116.842 E | 30.043 N | 0.7101 | 0.000010 |
| AH-37 | Alluvium | 117.102 E | 30.199 N | 0.7114 | 0.000013 |
| AH-38 | Dolomite | 117.010 E | 30.087 N | 0.7118 | 0.000009 |
| AH-39 | Dolomite | 117.077 E | 30.189 N | 0.7120 | 0.000010 |
| AH-40 | Moyite | 117.762 E | 30.701 N | 0.7100 | 0.000012 |
| AH-41 | Conglomerate | 117.656 E | 30.682 N | 0.7105 | 0.000010 |
| AH-42 | Granodiorite | 117.785 E | 30.575 N | 0.7094 | 0.000011 |
| AH-43 | Monzonitic granite | 117.916 E | 30.580 N | 0.7097 | 0.000013 |
| AH-44 | Granite | 117.852 E | 30.493 N | 0.7099 | 0.000011 |
| AH-45 | Sandstone | 117.896 E | 30.728 N | 0.7102 | 0.000010 |
| AH-46 | Limestone | 117.971 E | 30.592 N | 0.7107 | 0.000012 |
| AH-47 | Sandstone | 119.172 E | 30.771 N | 0.7145 | 0.000012 |
| AH-48 | Sandstone | 119.316 E | 31.100 N | 0.7092 | 0.000012 |
| AH-49 | Mudstone | 119.554 E | 31.052 N | 0.7095 | 0.000009 |
| AH-50 | Sandstone | 118.150 E | 30.477 N | 0.7128 | 0.000012 |
| AH-51 | Mudstone | 118.447 E | 30.629 N | 0.7091 | 0.000012 |
| AH-52 | Monzonitic granite | 118.451 E | 30.462 N | 0.7109 | 0.000011 |
| AH-53 | Sandstone | 118.527 E | 30.608 N | 0.7126 | 0.000009 |
| AH-54 | Shale | 118.460 E | 30.358 N | 0.7126 | 0.000010 |
| AH-55 | Dolomite | 118.595 E | 30.055 N | 0.7104 | 0.000009 |
| AH-56 | Granite | 118.728 E | 30.131 N | 0.7126 | 0.000008 |
| AH-57 | Granodiorite | 119.285 E | 30.385 N | 0.7099 | 0.000011 |
| AH-58 | Dolomite | 119.139 E | 30.409 N | 0.7110 | 0.000011 |

| | | | | | |
|---|---|---|---|---|---|
| AH-59 | Alluvium | 118.907 E | 30.714 N | 0.7088 | 0.000006 |
| AH-60 | Conglomerate | 118.700 E | 30.963 N | 0.7135 | 0.000011 |
| AH-61 | Glacial deposits | 118.983 E | 30.973 N | 0.7095 | 0.000012 |
| AH-62 | Alluvium | 118.785 E | 30.941 N | 0.7105 | 0.000010 |
| AH-63 | Alluvium | 118.788 E | 31.193 N | 0.7087 | 0.000007 |
| AH-64 | Moyite | 118.142 E | 30.201 N | 0.7114 | 0.000012 |
| AH-65 | Granodiorite | 118.280 E | 29.966 N | 0.7123 | 0.000014 |
| AH-66 | Sandstone | 117.687 E | 30.030 N | 0.7143 | 0.000011 |
| AH-67 | Shale | 117.602 E | 30.007 N | 0.7144 | 0.000014 |
| AH-68 | Shale | 117.602 E | 30.007 N | 0.7147 | 0.000008 |
| AH-69 | Tuff | 117.599 E | 29.992 N | 0.7152 | 0.000010 |
| AH-70 | Sandstone | 117.736 E | 29.921 N | 0.7160 | 0.000012 |
| AH-71 | Slate | 117.477 E | 29.872 N | 0.7170 | 0.000011 |
| AH-72 | Conglomerate | 117.775 E | 29.932 N | 0.7187 | 0.000012 |
| AH-73 | Granodiorite | 117.580 E | 30.049 N | 0.7116 | 0.000010 |
| AH-74 | Shale | 118.502 E | 29.972 N | 0.7127 | 0.000013 |
| AH-75 | Ophiolite | 118.599 E | 29.923 N | 0.7135 | 0.000011 |
| AH-76 | Breccia | 118.763 E | 30.023 N | 0.7163 | 0.000010 |
| AH-77 | Monzonite | 118.442 E | 29.629 N | 0.7191 | 0.000011 |
| AH-78 | Conglomerate | 118.416 E | 29.647 N | 0.7216 | 0.000011 |
| AH-79 | Ophiolite | 118.474 E | 29.861 N | 0.7115 | 0.000012 |
| AH-80 | Sandstone | 118.116 E | 29.791 N | 0.7138 | 0.000011 |
| AH-81 | Sandstone | 118.249 E | 29.733 N | 0.7101 | 0.000011 |
| AH-82 | Conglomerate | 118.032 E | 29.809 N | 0.7109 | 0.000011 |
| AH-83 | Dolomite | 117.984 E | 29.879 N | 0.7130 | 0.000011 |
| AH-84 | Conglomerate | 118.364 E | 29.753 N | 0.7127 | 0.000011 |
| AH-85 | Conglomerate | 115.920 E | 32.273 N | 0.7134 | 0.000011 |
| AH-86 | Diorite | 116.321 E | 31.359 N | 0.7149 | 0.000011 |
| AH-87 | Gneiss | 116.002 E | 31.175 N | 0.7091 | 0.000013 |
| AH-88 | Spilite | 116.165 E | 31.418 N | 0.7097 | 0.000011 |
| AH-89 | Siltite | 116.451 E | 31.388 N | 0.7101 | 0.000013 |
| AH-90 | Monzonitic granite | 115.498 E | 31.504 N | 0.7093 | 0.000011 |
| AH-91 | Gneiss | 115.801 E | 31.197 N | 0.7097 | 0.000011 |

| | | | | | |
|---|---|---|---|---|---|
| AH-92 | Syenite | 116.103 E | 31.597 N | 0.7101 | 0.000011 |
| AH-93 | Conglomerate | 116.009 E | 31.757 N | 0.7104 | 0.000012 |
| AH-94 | Granite | 115.723 E | 31.619 N | 0.7113 | 0.000012 |
| AH-95 | Sandstone | 115.964 E | 31.676 N | 0.7121 | 0.000010 |
| AH-96 | Sandstone | 116.587 E | 31.193 N | 0.7091 | 0.000011 |
| AH-97 | Gneiss | 116.637 E | 31.340 N | 0.7094 | 0.000011 |
| AH-98 | Alluvium | 117.150 E | 31.508 N | 0.7104 | 0.000013 |
| AH-99 | Phyllite | 116.622 E | 31.187 N | 0.7121 | 0.000012 |
| AH-100 | Sandstone | 118.012 E | 30.821 N | 0.7086 | 0.000006 |
| AH-101 | Sandstone | 118.162 E | 30.964 N | 0.7102 | 0.000011 |
| AH-102 | Sandstone | 118.065 E | 30.835 N | 0.7132 | 0.000004 |
| AH-103 | Alluvium | 118.398 E | 31.057 N | 0.7115 | 0.000007 |
| AH-104 | Conglomerate | 118.091 E | 30.813 N | 0.7094 | 0.000009 |
| AH-105 | Conglomerate | 116.620 E | 30.732 N | 0.7115 | 0.000012 |
| AH-106 | Sandstone | 116.272 E | 30.224 N | 0.7101 | 0.000012 |
| AH-107 | Basalt | 116.109 E | 30.058 N | 0.7104 | 0.000013 |
| AH-108 | Alluvium | 116.298 E | 30.191 N | 0.7106 | 0.000011 |
| AH-109 | Shallow particle rock | 115.942 E | 30.379 N | 0.7120 | 0.000011 |
| AH-110 | Diorite | 116.731 E | 31.030 N | 0.7080 | 0.000011 |
| AH-111 | Orthophyre | 116.878 E | 31.043 N | 0.7098 | 0.000010 |
| AH-112 | Gneiss | 116.801 E | 31.040 N | 0.7115 | 0.000011 |
| AH-113 | Monzonite | 117.022 E | 30.728 N | 0.7086 | 0.000010 |
| AH-114 | Sandstone | 116.969 E | 30.593 N | 0.7088 | 0.000011 |
| AH-115 | Sandstone | 116.967 E | 30.627 N | 0.7090 | 0.000013 |
| AH-116 | Alluvium | 117.074 E | 30.611 N | 0.7090 | 0.000013 |
| AH-117 | Sandstone | 116.977 E | 30.662 N | 0.7082 | 0.000011 |
| AH-118 | Diorite | 117.003 E | 30.619 N | 0.7083 | 0.000011 |
| AH-119 | Gneiss | 116.402 E | 31.077 N | 0.7073 | 0.000012 |
| AH-120 | Granite | 116.354 E | 30.880 N | 0.7086 | 0.000011 |
| AH-121 | Monzonite | 116.185 E | 30.999 N | 0.7087 | 0.000013 |
| AH-122 | Granite | 116.359 E | 31.031 N | 0.7098 | 0.000011 |
| AH-123 | Gneiss | 116.260 E | 30.723 N | 0.7100 | 0.000011 |

| | | | | | |
|---|---|---|---|---|---|
| AH-124 | Sandstone | 116.689 E | 30.473 N | 0.7107 | 0.000013 |
| AH-125 | Keratophyre | 116.319 E | 30.344 N | 0.7093 | 0.000012 |
| AH-126 | Gneiss | 116.349 E | 30.376 N | 0.7102 | 0.000011 |
| AH-127 | Alluvium | 115.466 E | 33.539 N | 0.7116 | 0.000012 |
| AH-128 | Alluvium | 115.466 E | 33.539 N | 0.7118 | 0.000011 |
| AH-129 | Eolian deposit | 115.648 E | 33.214 N | 0.7122 | 0.000011 |
| AH-130 | Alluvium | 115.779 E | 32.734 N | 0.7116 | 0.000012 |
| AH-131 | Limestone | 117.005 E | 32.610 N | 0.7112 | 0.000010 |
| AH-132 | Limestone | 116.826 E | 32.603 N | 0.7127 | 0.000010 |
| AH-133 | Alluvium | 117.168 E | 32.622 N | 0.7117 | 0.000010 |
| AH-134 | Porphyry | 116.921 E | 34.158 N | 0.7129 | 0.000011 |
| AH-135 | Shale | 117.011 E | 34.053 N | 0.7137 | 0.000013 |
| AH-136 | Alluvium | 116.625 E | 34.313 N | 0.7114 | 0.000013 |
| AH-137 | Shale | 116.929 E | 34.201 N | 0.7116 | 0.000011 |
| AH-138 | Dolomite | 117.062 E | 34.037 N | 0.7123 | 0.000011 |
| AH-139 | Alluvium | 117.980 E | 31.035 N | 0.7097 | 0.000008 |
| AH-140 | Sandstone | 117.979 E | 30.854 N | 0.7083 | 0.000009 |
| AH-141 | Granodiorite | 118.015 E | 30.881 N | 0.7087 | 0.000007 |
| AH-142 | Siltite | 118.071 E | 30.894 N | 0.7085 | 0.000007 |
| AH-143 | Sandstone | 118.084 E | 30.953 N | 0.7100 | 0.000006 |
| AH-144 | Alluvium | 117.915 E | 30.976 N | 0.7102 | 0.000008 |
| AH-145 | Breccia | 117.960 E | 30.920 N | 0.7085 | 0.000005 |
| AH-146 | Mudstone | 117.926 E | 30.864 N | 0.7085 | 0.000008 |
| AH-147 | Alluvium | 116.575 E | 33.217 N | 0.7124 | 0.000012 |
| AH-148 | Alluvium | 115.938 E | 33.730 N | 0.7124 | 0.000012 |
| AH-149 | Gneiss | 117.192 E | 32.945 N | 0.7106 | 0.000012 |

**Supplementary Table S2** Sample information of water.

| Sample Number | Longitude | Latitude | $^{87}Sr/^{86}Sr$ | 2σ |
|---|---|---|---|---|
| QTH5 | 117.881 E | 30.508 N | 0.7094 | NA |
| LYH10 | 117.906 E | 30.482 N | 0.7096 | NA |
| QTH6 | 117.885 E | 30.525 N | 0.7097 | NA |
| QTH2 | 117.882 E | 30.479 N | 0.7099 | NA |

| | | | | |
|---|---|---|---|---|
| QTH1 | 117.876 E | 30.468 N | 0.7102 | NA |
| LYH8 | 117.872 E | 30.454 N | 0.7103 | NA |
| QTH4 | 117.877 E | 30.499 N | 0.7103 | NA |
| LYH9 | 117.852 E | 30.402 N | 0.7103 | NA |
| LYH7 | 117.873 E | 30.448 N | 0.7103 | NA |
| QTH3 | 117.880 E | 30.491 N | 0.7102 | NA |
| CJ11 | 116.955 E | 30.442 N | 0.7107 | 0.000008 |
| ZGH-5 | 117.863 E | 31.787 N | 0.7100 | 0.000013 |
| ZGH-24 | 117.668 E | 31.684 N | 0.7100 | 0.000017 |
| ZGH-4 | 117.887 E | 31.788 N | 0.7101 | 0.000016 |
| ZGH-2 | 117.888 E | 31.773 N | 0.7101 | 0.000014 |
| ZGH-11 | 117.789 E | 31.764 N | 0.7108 | 0.000017 |
| ZGH-14 | 117.792 E | 31.623 N | 0.7110 | 0.000016 |
| ZGH-1 | 117.825 E | 31.691 N | 0.7110 | 0.000013 |
| ZGH-17 | 117.641 E | 31.655 N | 0.7111 | 0.000014 |
| ZGH-21 | 117.631 E | 31.679 N | 0.7111 | 0.000014 |
| ZGH-16 | 117.681 E | 31.663 N | 0.7111 | 0.000014 |
| ZGH-13 | 117.788 E | 31.632 N | 0.7112 | 0.000014 |
| ZGH-18 | 117.636 E | 31.658 N | 0.7112 | 0.000014 |
| ZGH-12 | 117.790 E | 31.628 N | 0.7112 | 0.000014 |
| ZGH-15 | 117.791 E | 31.625 N | 0.7113 | 0.000016 |
| ZGH-19 | 117.635 E | 31.662 N | 0.7113 | 0.000017 |
| ZGH-20 | 117.634 E | 31.674 N | 0.7114 | 0.000017 |
| ZGH-6 | 117.675 E | 31.821 N | 0.7115 | 0.000014 |
| ZGH-23 | 117.674 E | 31.677 N | 0.7118 | 0.000017 |
| ZGH-8 | 117.708 E | 31.845 N | 0.7120 | 0.000014 |
| ZGH-10 | 117.763 E | 31.767 N | 0.7120 | 0.000017 |
| ZGH-22 | 117.675 E | 31.672 N | 0.7125 | 0.000019 |
| ZGH-7 | 117.655 E | 31.792 N | 0.7125 | 0.000016 |
| ZGH-9 | 117.732 E | 31.858 N | 0.7126 | 0.000014 |
| ZGH-25 | 117.668 E | 31.687 N | 0.7126 | 0.000017 |
| ZGH-3 | 117.884 E | 31.800 N | 0.7102 | 0.000014 |
| YZ-DT-M* | 117.732 E | 30.841 N | 0.7107 | 0.000005 |

| | | | | |
|---|---|---|---|---|
| YZ-DT-S* | 117.732 E | 30.841 N | 0.7107 | 0.000004 |
| YZ-DT-B* | 117.732 E | 30.841 N | 0.7107 | 0.000003 |
| CJ10 | 117.713 E | 30.796 N | 0.7107 | 0.000011 |
| CJ08 | 118.417 E | 31.623 N | 0.7107 | 0.000014 |
| CJ12 | 116.252 E | 29.796 N | 0.7107 | 0.00001 |
| PYH | 116.351 E | 29.861 N | 0.7144 | 0.000005 |
| CJ-44 | 116.542 E | 29.903 N | 0.7110 | NA |
| CJ09 | 118.021 E | 31.225 N | 0.7108 | 0.000009 |

**Supplementary Table S3** Anhui Province geographical indication products, trademarks and agricultural products used for traceability cases.

| Category | Products | Territory | Enactment Time |
|---|---|---|---|
| Geographical indication agricultural products | Yuexi Cuilan | Yuexi County, Anqing City | 2021 |
| Geographical indication agricultural products | Tongcheng Xiaohua | Tongcheng City, Anqing City | 2018 |
| Geographical indication agricultural products | Shitai Xiangya | Shitai County, Chizhou City | 2011 |
| Geographical indication agricultural products | Dudu Cuiming | Chaohu City, Hefei City | 2018 |
| Geographical indication agricultural products | Taiping Houkui | Huangshan District, Huangshan City | 2021 |
| Geographical indication products | Ancha | Qimen County, Huangshan City | 2013 |
| Geographical indication agricultural products | Huoshan Huangya | Huoshan County, Luan City | 2018 |
| Geographical indication products | Hanmei Lvcha | Hanshan County, Maanshan City | 2018 |
| Geographical indication agricultural products | Yongxi Huoqing | Jingxian County, Xuancheng City | 2011 |
| Geographical indication agricultural products | Huanghua Yunjian | Ningguo City, Xuancheng City | 2019 |

| | | | |
|---|---|---|---|
| Geographical indication agricultural products | Lixing Jiegeng | Taihe County, FuyangCity | 2021 |
| Geographical indication trademark | Tongcheng Jiegeng | Tongcheng City, Anqing City | 2020 |
| Geographical indication trademark | Jinzhai Huangjing | Jinzhai County, Luan City | 2020 |
| Geographical indication agricultural products | Jiuhua Huangjing | Qingyang County, Chizhou City | 2017 |
| Geographical indication trademark | Qimen Huangjing | Qimen County, Huangshan City | 2024 |
| Geographical indication agricultural products | Bodanpi | Qiaocheng District, Bozhou City | 2017 |
| Geographical indication products | Fengdanpi | Yian District, Tongling City and Nanling County, Wuhu City | 2006 |
| Geographical indication agricultural products | Xuanmugua | Xuanzhou District, Xuancheng City | 2010 |
